# Genetic Loci Conferring Reducing Sugar Accumulation and Conversion of Cold-Stored Potato Tubers Revealed by QTL Analysis in a Diploid Population

**DOI:** 10.3389/fpls.2018.00315

**Published:** 2018-03-09

**Authors:** Guilin Xiao, Wei Huang, Hongju Cao, Wei Tu, Haibo Wang, Xueao Zheng, Jun Liu, Botao Song, Conghua Xie

**Affiliations:** ^1^Key Laboratory of Potato Biology and Biotechnology, Ministry of Agriculture, Wuhan, China; ^2^National Center for Vegetable Improvement (Central China), Wuhan, China; ^3^Potato Engineering and Technology Research Center of Hubei Province, Wuhan, China; ^4^College of Horticulture and Forestry Sciences, Huazhong Agricultural University, Wuhan, China; ^5^Key Laboratory of Horticultural Plant Biology (HZAU), Ministry of Education, Wuhan, China; ^6^College of Life Science and Technology, Huazhong Agricultural University, Wuhan, China

**Keywords:** potato, QTL, cold-induced sweetening, recondition, cumulative effects

## Abstract

Cold-induced sweetening (CIS) caused by reducing sugar (RS) accumulation during storage in low temperature in potato tubers is a critical factor influencing the quality of fried potato products. The reconditioning (REC) by arising storage temperature is a common measure to lower down RS. However, both CIS and REC are genotype-dependent and the genetic basis remains uncertain. In the present study, with a diploid potato population with broad genetic background, four reproducible QTL of CIS and two of REC were resolved on chromosomes 5, 6, and 7 of the CIS-sensitive parent and chromosomes 5 and 11 of the CIS-resistant parent, respectively, implying that these two traits may be genetically independent. This hypothesis was also supported by the colocalization of two functional genes, a starch synthesis gene *AGPS2* mapped in QTL CIS_E_07-1 and a starch hydrolysis gene *GWD* colocated with QTL REC_B_05-1. The cumulative effects of identified QTL were proved to contribute largely and stably to CIS and REC and confirmed with a natural population composed of a range of cultivars and breeding lines. The present research identified reproducible QTL for CIS and REC of potato in diverse conditions and elucidated for the first time their cumulative genetic effects, which provides theoretical bases and applicable means for tuber quality improvement.

## Introduction

Potato (*Solanum tuberosum* L.), with a world production of about 377 million tons in 2016^1^ play an important role in food security in the world as it can be consumed as both staple food and processed products. For processing, potato tubers are often stored in low temperature to reduce loss from sprouting, shrinking, and pathogenesis. However, exposed to low temperature for a certain period usually results in an accumulation of reducing sugars (RSs) in the tubers, a phenomenon known as cold-induced sweetening (CIS). A nonenzymatic Maillard reaction (Maillard, 1912) between RS and α-amino acids of nitrogenous compounds during frying in high temperature leads to dark-colored and bitter products, and increasingly being concerned, the formation of acrylamide which is likely a carcinogen (Mottram et al., 2002). The RS content can fall back to a low level through reconditioning (REC) before processing by increasing the storage temperature to above 20°C, which allows a partial RS conversion to starch, or to be used by respiration (Illeperuma et al., 1998). However, recondition is costly and insufficient since the REC capacity varies among potato genotypes, possibly the cause for most varieties undesirable for frying though they have low RS content at harvest.

Much attention was initially paid on the physiology and biochemistry of CIS involved in the interconversion of starch and sugars. This conversion is thought to be affected by starch breakdown and synthesis, sucrose metabolism and transport, glycolysis, as well as mitochondrial respiration (Sowokinos, 2001). In recent years, substantial molecular evidences have revealed carbohydrate metabolism involved in potato CIS. The most in-depth study is sucrose pathway in which the vacuolar acid invertase StvacINV1 and its specific inhibitor StInvInh2B play crucial roles in sucrose conversion to RSs (Liu et al., 2010, 2011, 2013a,b). Subsequent experiments have suggested that the inhibition of StvacINV1 by StInvInh2B is blocked by SbSnRK1β and restored by phosphorylated form of SbSnRK1α (Lin et al., 2013, 2015). Besides, several enzymes function in starch metabolism has been proved functional in CIS by reverse genetic approaches, such as α-glucan, water dikinase (GWD) and starch phosphorylase L gene catalyzing the phosphorylation of starch (Lorberth et al., 1998; Mikkelsen et al., 2005; Kamrani et al., 2011), SbAI, and StAmy23 which are α-amylase as well as β-amylase StBAM1 and StBAM9 for starch hydrolysis by acting on different substrates in different subcellular locations (Zhang et al., 2014; Hou et al., 2017). Most recently, the glyceraldehyde-3-phosphate dehydrogenase (GAPDH) genes *StGAPC1*, *StGAPC2*, and *StGAPC3* are demonstrated to regulate potato CIS by altering carbohydrate pools toward sucrose synthesis and cleavage rather than glycolysis (Liu et al., 2017). However, the genetic basis of potato CIS is still unclear, also, little information is available for REC, which hamper the improvement of tuber processing quality.

To elucidate the genetic mechanism of CIS, the phenotypic variation in chip color has been deciphered by quantitative traits loci (QTL) analysis with different segregating populations. The first mapping was carried out with a diploid potato population (*Solanum* spp.) which identified six QTL on chromosomes 2, 4, 5, and 10 for chip color of the tubers stored at 10°C for 45 days (d) (Douches and Freyre, 1994). QTL for chipping color of cold-stored tubers were identified in other two diploid populations, one was C × E (US-W5337.3 × 77.2102.37) with QTL located on chromosomes 3, 5, 8, and 10 (Werij et al., 2012) and the other was 11–40 (DG 97-952 × DG 08-26/39) with QTL located only on chromosome 6. Notably, two parents of the latter population were multigenerational *Solanum* hybrids originating from 10 potato species (Sołtys-Kalina et al., 2015). The QTL for fry color of potato tubers after cold storage were identified on chromosomes 6 and 9 in a tetraploid potato population (*Solanum tuberosum* subsp. *tuberosum*) (Bradshaw et al., 2008). The only report considering sugar content of cold stored tubers revealed that QTL for sucrose and RS content were located on all potato chromosomes in a diploid mapping population descending from *S. tuberosum* (Menéndez et al., 2002), suggesting an extreme complexity of carbohydrate metabolism of potato tubers in response to environments. As is well known, sugars can be converted to starch with varied levels when potato tubers are transferred from cold storage to warmer conditions (above 20°C), which is usually accompanied by an increase in respiration rate (Isherwood, 1973; Nourian et al., 2003). Nevertheless, there are few studies about the genetic basis of REC. Werij et al. (2012) mapped QTL for chipping color after recondition on chromosomes 5 and 10 in a diploid population. Within a different diploid population, QTL were differently located, on chromosomes 1 and 6 (Sołtys-Kalina et al., 2015).

Although the candidate gene approach colocalized a number of functional genes in carbohydrate metabolism together with or linked to the QTL for sugar content, such as the genes encoding invertase, sucrose synthase 3, sucrose phosphate synthase, ADP-glucose pyrophosphorylase, etc. (Menéndez et al., 2002), genetic dissection of CIS and REC could not be cogent for these complex traits without involvement of more segregating populations descended from as wider as possible genetic backgrounds.

Here, we report the reproducible QTL contributing to the CIS and the sugar conversion by REC of a wide genetic background potato diploid population. We find for the first time that cumulative effects across reproducible QTL play crucial roles in tuber CIS and REC, these effects on CIS were validated in a natural population (NP) suggesting the potentials of the linked markers used for potato quality breeding.

## Materials and Methods

### Plant Materials

The diploid potato segregating population referred as EB population consisting of 178 individuals was used for QTL mapping. The population was derived from the cross between CIS-sensitive diploid clone ED25 (E) as the maternal parent and CIS-resistant diploid wild species *Solanum berthaultii* acc. CW2-1 (B) as the paternal parent. ED25 is a hybrid descending from the cross of diploid clone 77.2102.37 which bearing genetic backgrounds of *S*. *phureja*, *S*. *tuberosum*, and *S*. *vernei* and USW7589.2 (*S*. *tuberosum* × *S*. *phureja*) (Jacobsen, 1980).

Additional 89 varieties and advanced breeding lines (*S*. *tuberosum*) referred to as NP with different levels of CIS resistance (Supplementary Table S1) were employed for evaluating the cumulative effects of the QTL identified in this study.

### Assessment of Reducing Sugar Content

All potato genotypes, i.e., the parents and individuals of the EB population, were grown in four geographically diverse sites where potato is taken as one of the main crops in between 2008 and 2012 (Supplementary Table S2). Each field trial, carried out in different site or year, was considered as an independent environment numerically numbered. Randomized block design with triplication was used, and each block contained 10 plants per individual. The field management was conducted according to local practices to ensure a normal crop growth. Potato tubers were harvested when plants presented foliage senescence and kept in room temperature (20–22°C) for 7 d to allow skin set before stored at 4°C for 30 d, then reconditioned for 20 d at 20–22°C. The tubers from each block were stored separately, sampled as independent biological replicates for biochemical analysis. Three-to-five tubers were sampled per replicate. For RS measurement, samples were taken from two ends of the tuber (the stem and the bud ends) and the middle with a hole punch, sliced, combined, freeze dried, and ground to a fine powder. RSs were extracted with 80% (v/v) aqueous ethyl alcohol from 10 to 15 mg freeze-dried ground tissue for 1 h at 80°C and measured by the 3,5-dinitrosalicylic acid method (Lindsay, 1973).

The RS content of tubers after storage at 4°C for 30 d reflects the response of a potato genotype to low temperature and this trait is nominated CIS. The RS content after REC represents the capacity of a potato genotype to turn over the RS accumulated in low temperature to starch or for increasing respiratory consumption which was nominated REC. Both CIS and REC were used as target traits for QTL mapping in the present research.

The potato varieties and advanced breeding lines of the NP population were grown in pots with normal management under greenhouse conditions in Huazhong Agricultural University, Wuhan in 2013. Four pots of each genotype were harvested and the tubers were treated as above for sampling and CIS phenotyping after stored at 4°C for 30 days. These data were used for evaluating the cumulative effects of the identified QTL.

### Molecular Marker Assays

Genomic DNA was extracted according to the CTAB protocol as described by Dellaporta et al. (1983). Molecular markers including 303 SSRs, 37 AFLP primer combinations, and 141 candidate gene markers (CGMs) (Menéndez et al., 2002; Gebhardt et al., 2005; Li et al., 2005, 2008, 2013; Werij et al., 2012) were used for polymorphic analysis between the parents of the EB population. Among the SSR markers tested, 204 were from Milbourne et al. (1998), Ghislain et al. (2001, 2006, 2009), and Feingold et al. (2005) while the rest were newly designed based on the potato genomic DNA sequence (PGSC v4.03) ^2^ as described by Chen et al. (2014) for densifying the chromosomes 5 and 6 which were considered possibly harboring reproducible QTL related to CIS in our preliminary study. The amplification of SSRs and CGMs was performed according to relevant publications. The PCR products were separated by 9% polyacrylamide gel electrophoresis and silver-stained. AFLP assays were carried out as described by Vos et al. (1995). Primer sequences for pre-amplification of *Eco*R I and *Mse* I were 5′-GACTGCGTACCAATTC-3′ and 5′-GATGAGTCCTGAGTAA-3′ and the PCR products were separated by 6% denatured polyacrylamide gels. The polymorphic SSRs, CGMs, and AFLP primer pairs were used for genotyping the entire EB population.

### Map Construction, QTL Mapping, and Marker Validation

All potato genotypes were determined manually after electrophoresis of the PCR products, with each present band being scored as a dominant versus recessive (absent) allele. The name of each band consisted of primer name suffixed by band size estimated based on DNA standard ΦX174 HaeIII Digest (NEB).

A pseudo-testcross strategy was adopted to construct linkage map for each parent using software Joinmap^®^4 (Van Ooijen, 2006) with the following settings: CP population type, independence logarithm of the odds (LOD) as a grouping parameter (linkages with LOD > 3 were considered significant), regression mapping algorithm, and Kosambi’s mapping function. The number of linkage groups and the orientations were determined by SSRs and CGMs in the references.

Distribution of RS contents representing CIS and REC of the EB population was analyzed for each growth environment and the correlations between environments were assumed by SPSS18.0 (IBM, New York, NY, United States). The QTL analysis was separately conducted with linkage map for each parent with software package MapQTL^®^6 based on multiple-QTL models (MQM) (Van Ooijen, 2009). Detection of QTL was done after 1,000 permutations, the genome-wide threshold (*p* = 0.05) for each trait was identified and the highest LOD threshold was chosen to utilize as the final threshold. The two-LOD support interval was taken as a confidence interval for each QTL. When a peak LOD exceeded the threshold, a QTL was declared and named with trait name CIS or REC and the chromosome location. If two-LOD support intervals of two QTL overlapped each other, the QTL were considered as the same QTL with same name unless they were detected for different traits. The linkage maps and the positions of QTL were drawn by MapChart 2.2 (Voorrips, 2002).

## Results

### Reducing Sugar Content Distribution of EB Population

**FIGURE 1:**
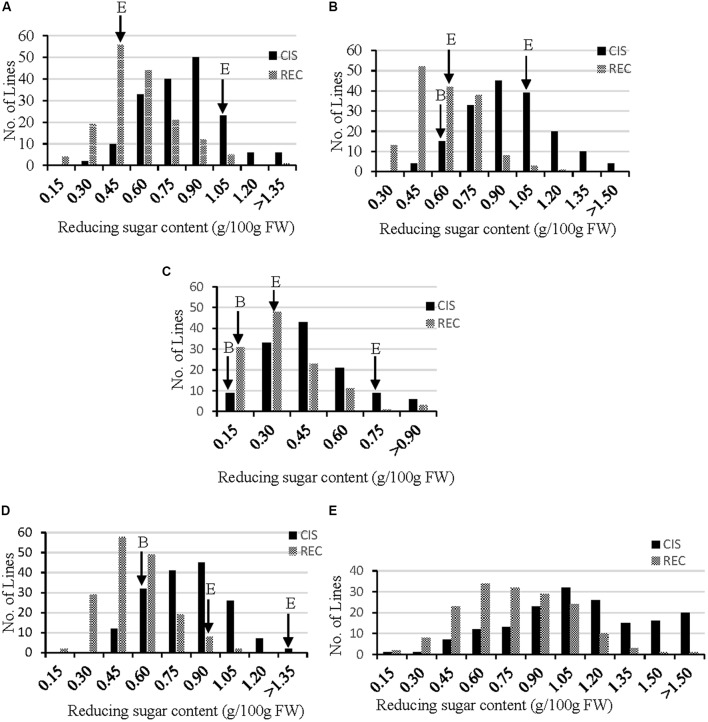
Frequency distribution of reducing sugar (RS) content for CIS (black bar, stored at 4°C for 30 d) and REC (gray bar, stored at 4°C for 30 d and then reconditioned at 20°C for 20 d) in the EB population in five environments (**A–E**, representing environment 1 to 5 refer to Supplementary Table S2 for the site detail). E and B shorts for CIS-sensitive and CIS-resistance parent, respectively. Some data of parents are missing due to the shortage of tubers.

In order to dissect the genetic loci controlling RSs related to the traits of CIS (RS after storage at 4°C for 30 d) and REC (RS after storage at 4°C for 30 d followed by 20 d of REC at 20°C), the RS content was measured for all genotypes grown under five different environments (Supplementary Table S2). Distribution of CIS and REC fitted well with normal curve (**Figure 1**), suggesting both CIS and REC are quantitative traits that controlled by multigenes. The paternal parent B, an accession of *S*. *berthaultii*, showed lower CIS and REC than the maternal parent E (**Figure 1**), indicating a higher CIS resistance level and a stronger REC capacity of the former than the later. Moreover, both CIS and REC were significantly correlated between the environments (*p* < 0.05) suggesting these two traits are largely genotype-dependent although some variations may exist as reflected by lower (but still significant) correlation coefficients (**Table 1**). These phenotyping data were used for QTL analysis of CIS and REC.

**Table 1:** Correlation coefficients of RSs between the growing environments measured for CIS and REC of the EB population.

Trait^a^	Trait	CIS					REC				
	Environment code^b^	1	2	3	4	5	1	2	3	4	5
CIS	1	1	0.686^∗∗^	0.446^∗∗^	0.760^∗∗^	0.443^∗∗^	0.798^∗∗^	0.681^∗∗^	0.427^∗∗^	0.628^∗∗^	0.367^∗∗^
	2		1	0.299^∗∗^	0.594^∗∗^	0.301^∗∗^	0.584^∗∗^	0.705^∗∗^	0.301^∗∗^	0.487^∗∗^	0.365^∗∗^
	3			1	0.233^∗^	0.303^∗∗^	0.368^∗∗^	0.277^∗∗^	0.558^∗∗^	0.247^∗∗^	0.190^∗^
	4				1	0.465^∗∗^	0.590^∗∗^	0.541^∗∗^	0.224^∗^	0.760^∗∗^	0.427^∗∗^
	5					1	0.431^∗∗^	0.433^∗∗^	0.247^∗∗^	0.448^∗∗^	0.148
REC	1						1	0.779^∗∗^	0.378^∗∗^	0.763^∗∗^	0.374^∗∗^
	2							1	0.317^∗∗^	0.709^∗∗^	0.349^∗∗^
	3								1	0.216^∗^	0.255^∗∗^
	4									1	0.363^∗∗^
	5										1

*^a^CIS, RS content tested after storage at 4°C for 30 d; REC, RS content tested after storage at 4°C for 30 d and then reconditioned at 20°C for 20 d*.

*^b^Refer to Supplementary Table S2 for details. ^∗^*p* < 0.05, ^∗∗^*p* < 0.01*.

### Genetic Linkage Maps

A total of 303 SSR markers and 141 CGMs were screened on the parents to identify polymorphic markers. Finally, 452 SSR bands generated by 169 pairs of SSR primers, 125 CGM bands by 55 pairs of the gene-specific primers, and 221 AFLP bands by 37 AFLP primer combinations were shown polymorphic and used for constructing the linkage map separately for parent B and E by two-way pseudo-testcross due to the lack of bridge markers descending from both parents simultaneously. The genetic map of parent B consisted of 12 linkage groups which were assigned to 12 chromosomes with 129 markers covering a total length of 1037.9 cM (Supplementary Figure S1A). The average distance between markers was 8.0 cM with the maximum interval of 35.8 cM on the top of chromosome 10. Averagely, there were 11 markers per chromosome with the most (19 markers) and least (4 markers) on chromosomes 3 and 1, respectively.

Compared to the paternal map, the maternal map had a higher density with 245 polymorphic markers resolved to 12 linkage groups (Supplementary Figure S1B). The total length of the map was 1043.5 cM with average marker distance of 4.3 cM. Despite the maximum interval (27.5 cM) was presented on the south end of the chromosome 5 it contains the largest number of markers (20) while the chromosome 9 has the least (7). According to the map density, it is assumed that the maternal parent E may possess a higher heterozygosity than paternal parent B as more polymorphic markers were mapped in the former.

Candidate gene approach by converting part of the gene sequence into specific functional marker is an effective way to identify genes for target traits. In the present research, out of 141 genes selected from those involved in carbohydrate metabolism and transport, 39 were mapped in the two maps, 29 polymorphic bands generated by 18 genes in the paternal map (Supplementary Figure S1A), and 54 polymorphic bands generated by 29 genes in the maternal map (Supplementary Figure S1B).

### QTL of CIS and REC

There were nine QTL of CIS detected in six chromosomes in the paternal map and also nine in five chromosomes in the maternal map. These QTL explained the phenotypic variance ranging 4.0–12.4% (Supplementary Table S3). There were 7 and 11 QTL of REC detected in the paternal and maternal map, respectively, explaining the phenotypic variance ranging 4.9–12.0% (Supplementary Table S3). However, most of these QTL could be detected in only one of the five environments, implying that CIS and REC are the traits controlled by multi-genetic loci and their genetic effects are minor and largely affected by environmental factors. This kind of QTL was considered lapsable and would not be included in the following research because of their elusive influence on the traits. Therefore, to explore the loci playing stable roles in the processes of CIS and REC, the QTL detected in two or more environments were taken and used for further analysis in the present research.

**FIGURE 2:**
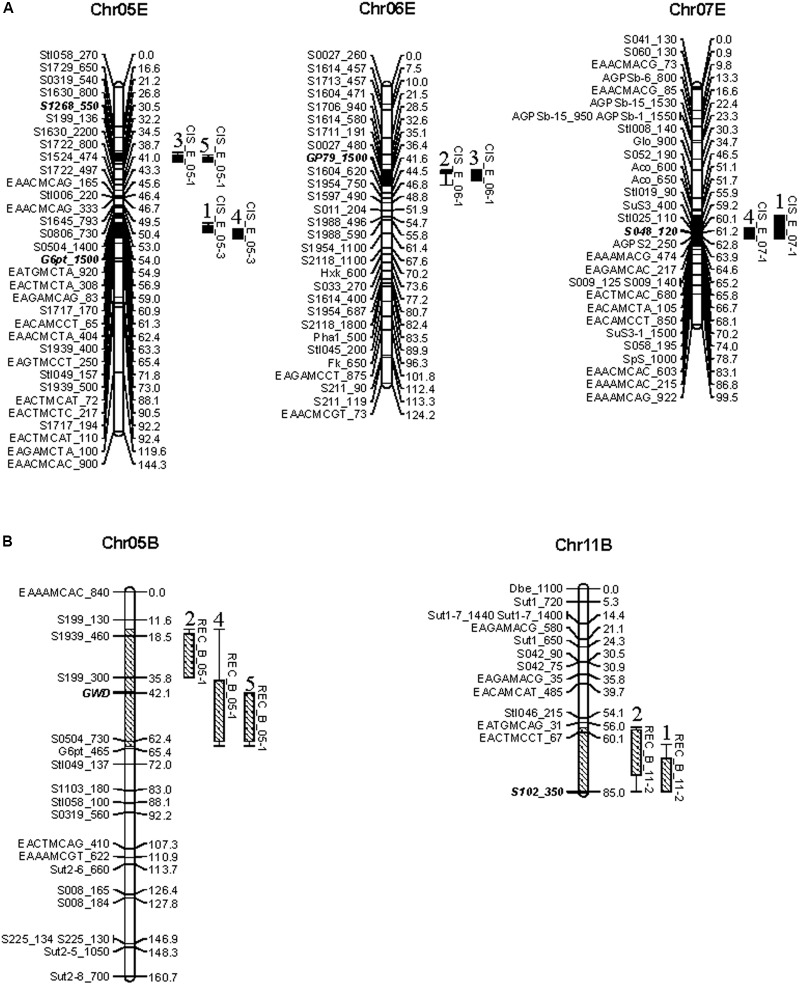
Reproducible QTL of CIS **(A)** and REC **(B)** was identified in two or more environments. On the top of each linkage group shows the number of chromosomes with B or E to denote CIS-resistance parent or CIS-sensitive parent. QTL labeled by CIS or REC and the chromosome number are indicated with –2 LOD confidence intervals. Solid and lower diagonal fill bars represent QTL for CIS and REC, respectively. On the top of the QTL is environment code. Markers for detecting cumulative effects across these QTL in EB and the NP are indicated as bold and italic.

There were four QTL for CIS distributed on chromosomes 5, 6, and 7 of parent E (**Figure 2A**) and two for REC on chromosomes 5 and 11 of parent B (**Figure 2B**). Obviously, the QTL of CIS and REC were, respectively, dissolved to the maps of CIS-sensitive and CIS-resistant parents, implying these two traits may be genetically independent. This finding was in accordance with different colocalized genes, the starch synthesis gene *AGPS2* was mapped in the region of QTL CIS_E_07-1 and the starch hydrolysis gene *GWD* was positioned together with QTL REC_B_05-1 (**Figure 2** and Supplementary Table S3).

### Cumulative Effects of QTL

**FIGURE 3:**
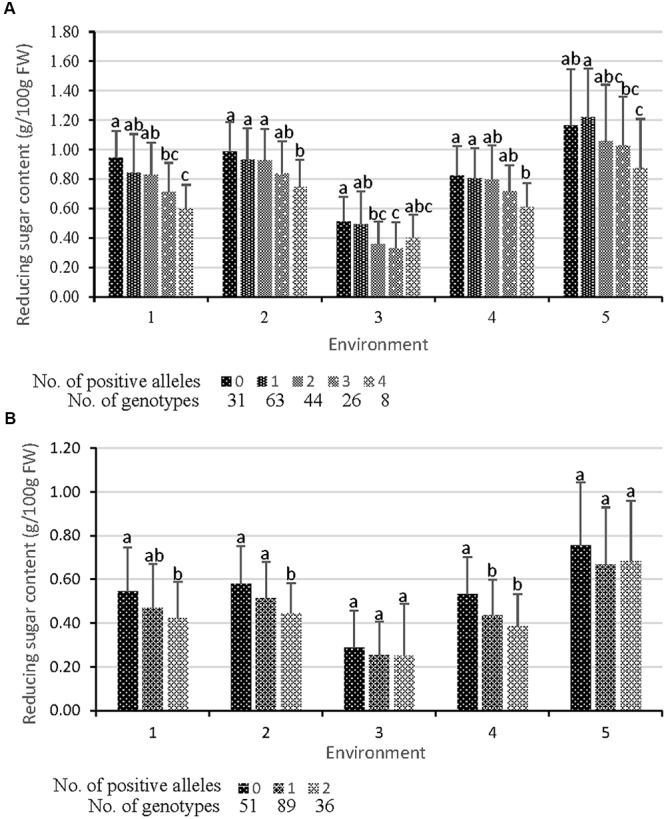
Cumulative effects of QTL. Each QTL is represented by a tightly linked marker showed in **Figure 2** as bold and italic font. **(A)** Effects on CIS (tuber RS content after cold storage at 4°C for 30 d). QTL CIS_E_05-1, CIS_E_05-3, CIS_E_06-1, and CIS_E_07-1 are represented by markers S1268_550, G6pt_1500, GP79_1500, and S048_120, respectively. **(B)** Effects on REC (tuber RS content reconditioned at 20°C for 20 d followed by the cold storage). Markers GWD and S102_350 represent QTL REC_B_05-1 and REC_B_11-2, respectively. The data for a given number of positive alleles were the mean values for all individuals having the positive alleles of the given number, whichever the positive alleles they were. Significant differences at *p* < 0.05 between each other were shown by different lower-case letters on the column.

To look further insight into the mechanism underlying the function of the QTL on tuber RS content, cumulative effects across identified QTL were analyzed in EB population and validated in the NP. The QTL of CIS and REC were represented by amplifying one tightly linked marker (showed in **Figure 2** as bold and italic font). Generally, individuals showing a decreasing trend in RS content contained more positive alleles in EB population under all five environments (**Figure 3** and Supplementary Table S4). Compared with the individuals that amplified no positive allele, the RS content of those with four positive alleles decreased by 21.9–36.6% for CIS (**Figure 3A**)and by 9.6–27.7% for REC (**Figure 3B**). There was a significant difference between them in four environments for CIS and three environments for REC (**Figure 3**). These results indicate that cumulative effects of the identified QTL may play a crucial role in controlling tuber CIS and REC.

The NP population composed of 89 potato cultivars and advanced breeding lines was employed to validate the cumulative effects for CIS. The RS content of the tubers stored at 4°C for 30 d was 0.59 g/100 g FW in average, ranging from 0.12 to 1.29 g/100 g FW (Supplementary Table S1). Seventeen genotypes with low RS content (<0.3 g/100 g FW) were considered as CIS resistant, and the other 66 genotypes with RS content of >0.3 g/100 g FW as CIS sensitive.

**FIGURE 4:**
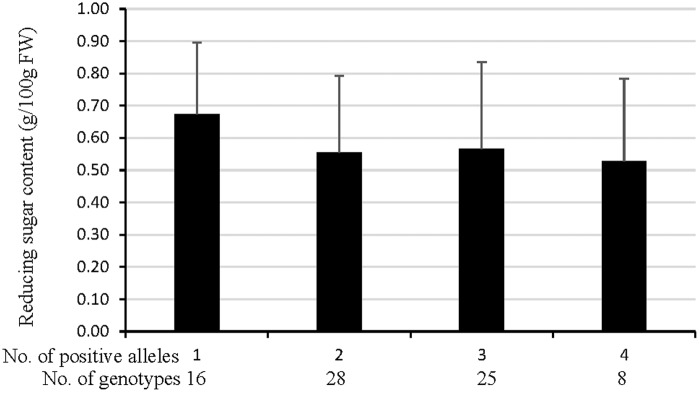
Validation of cumulative effects of markers that represented QTL (refer to **Figure 3** for details) for tuber RS content after cold storage at 4°C for 30 d in the NP. The data of genotypes that possessing none positive allele were missing because there was one genotype having none positive allele. The difference between each other showed not significant at *p* < 0.05.

The RS content in NP population gradually declined as the number of positive alleles increased (**Figure 4** and Supplementary Table S1). The mean RS content for genotypes possessing four positive alleles largely decreased by 21.6% although not showed a significant difference compared with that owning one positive allele (**Figure 4**), inferring that RS content after cold storage was largely regulated by cumulative effects of the identified QTL. It was also in line with the number of positive alleles of CIS-resistant and CIS-sensitive genotypes, more were present in the former than in the later (Supplementary Table S1). These results confirmed that the identified QTL have collaborative function on potato CIS though these effects have not been tested for REC of the NP population.

## Discussion

Control of CIS is of great economic importance for potato processing industry. Unfortunately, most potato varieties are not suitable for the processing owing to the difficulties of lowering down RSs accumulated during low temperature storage to an acceptable level. More investigations on the genetic basis of CIS and REC are complementary to and essential for the improvement of new varieties. Previous studies mapped chip color QTL in almost all chromosomes though the number of QTL varied with individual reports (Douches and Freyre, 1994; Menéndez et al., 2002; Bradshaw et al., 2008; Werij et al., 2012), strongly demonstrating the genetic complexity of CIS and REC observed in any of the reported segregating populations. In the present research, the tuber RS content was, respectively, measured after storage at 4°C for 30 d and REC at 20°C for 20 d for target traits of CIS and REC, which is more directly relevant to and more precise for the issues concerned in tuber quality. The identification of new QTL for CIS and REC in the present research further evidence that these two traits are controlled by multiple and minor genes since the QTL responsible for each trait distribute in at least five chromosomes with explanation to phenotypic variation of 4.0–12.4%. However, selection of reproducible QTL under diverse environments seems to be effective for studying such complex traits that affected largely by environmental factors. The positions of reproducible QTL correlate well with that of chip color (Douches and Freyre, 1994; Bradshaw et al., 2008; Werij et al., 2012) or sugar content (Menéndez et al., 2002) though strict comparison between them could not be made as a result of the shortage of common markers.

Colocalization of relevant genes suggests a reliable mapping of the QTL and provides a route for dissecting biological functions of the QTL. Totally, 39 functional genes in carbohydrate metabolism were located on two linkage maps (Supplementary Figure S1). Moreover, we have identified four CIS QTL and two REC QTL which include functional genes involved in carbohydrates metabolism colocalized or flanked (**Figure 2**). For example, the *AGPS2* gene positions in the QTL CIS_E_07-1, the *GWD* gene in the QTL REC_B_05-1, with *G6pt* gene nearby, and another *G6pt* allele locates north to the QTL CIS_E_05-3. AGPase is an enzyme composed of two subunits referred as AGPaseS and AGPaseB to catalyze G-1-P and ATP to form ADP-Glucose that is the sole source of starch synthesis (Tetlow et al., 2004). The locus of *AGPaseS* on chromosome 1 was proved not only colocalized with sugar or starch content QTL (Menéndez et al., 2002; Gebhardt et al., 2005), but also positively associated with chip color after cold storage at 4°C (Li et al., 2013). *AGPS2*, also the subunit AGPaseS, has been mapped on chromosome 7 in the research of Werij et al. (2012) but for the first time to colocalize with CIS QTL in the present research implying its contribution to this QTL and potential roles to CIS. The *GWD* gene is important for starch degradation (Ritter et al., 2002; Mikkelsen et al., 2005). The phosphate content of starch and RS content of potato tubers could be reduced by down-regulating *GWD* expression (Lorberth et al., 1998). This gene has been found in many cases to colocalize with the QTL conferring chip color of the cold-stored tubers (Douches and Freyre, 1994) and the tubers after REC (Werij et al., 2012), fructose, and glucose content (Menéndez et al., 2002), in a similar genetic region as we identified on chromosome 5 for REC and CIS (**Figure 2**). One *G6pt* allele also locate near these regions. G6pt is the member of the glucose 6-phosphate/phosphate translocators importing carbon skeletons in the form of Glc6P derived from sucrose into plastids for starch synthesis or the oxidative pentose phosphate pathways (Flügge et al., 2011). Our results speculate that potato chromosome 5 may possess important genomic regions regulating the biological processes of CIS and REC through starch phosphorylation pathway.

The present research also implied that potato CIS and REC may be genetically controlled by different systems since corresponding QTL were resolved to the maps of CIS-sensitive and CIS-resistant parents, respectively (**Figure 2**). In fact, a very few potato varieties are suitable for chips or fries, not only because they contain higher RSs, but also, in most cases, largely due to an inadequate conversion of the RSs accumulated during cold storage. However, there was a report about the correlation between chip color after 4°C storage and recondition and one QTL on chromosome 10 was identified responsible for both traits (Werij et al., 2012). More efforts are needed to dissect the genetic mechanism underlying CIS and REC.

The present research is the first to demonstrate that both the CIS and REC QTL showed cumulative effects on the trait variation (**Figures 3**, **4**), not only in bi-parental (EB population) but also in multi-parent population (NP population), which will provide a theoretical basis for marker-assisted selection of potato processing quality. Eventually, the markers linked to the QTL are polymorphic in either EB population or NP population composed of widely adopted varieties and advanced breeding lines, fitted well with the performance of the individuals for CIS and REC. The more the positive alleles a genotype harbors, the lower the RS content in the process of CIS or REC will be. Our results indicate that the identified QTL synergistically function on regulating the RS content of potato tubers exposed to low temperature and the RS conversion response to the REC. For one gene, additive effects, known as the breeding values, are the most important genetic variance component. For a quantitative trait, cumulative effects of two or more genetic factors are important to the phenotype variation but have rarely been mentioned. Our results provide also great potentials of the tightly linked markers with QTL for improving CIS and REC since the QTL were identified in a segregating population with broad genetic background possibly possessing the ancestors of these two traits of modern cultivars.

## Author Contributions

CX, BS, and JL conceived and conducted the experiments, processed the data, and revised the manuscript. GX designed the methods and experiments, carried out the experiments, analyzed the data, and wrote the manuscript. WH, HC, WT, HW, and XZ grown the plants and detected the phenotypes. WH and HC performed the genotype analysis. All authors read and approved the final manuscript.

## Conflict of Interest Statement

The authors declare that the research was conducted in the absence of any commercial or financial relationships that could be construed as a potential conflict of interest.

## Footnotes

1 http://www.fao.org/

2 http://solanaceae.plantbiology.msu.edu/pgsc_download.shtml
