# Supplementary material for: Genetic Loci Conferring Reducing Sugar Accumulation and Conversion of Cold-Stored Potato Tubers Revealed by QTL Analysis in a Diploid Population
Source: Front Plant Sci. 2018 Mar 9;9:315. doi: 10.3389/fpls.2018.00315 (PMC5854652; doi:10.3389/fpls.2018.00315)
Supplement: Supplementary file 1 [file Table_1.PDF]

Supplementary Table S1 Potato varieties and breeding lines used for evaluating the cumulative effects of stable QTL and reducing sugar content (g/100 FW) after storage at 4 °C for 30 d

Genotype	RS content (Means±SD)	CIS Resistance	No. of positive alleles[a]	RS content (Means±SD)[b]
08HE077-13	0.687±0.140	Sensitive	0	-
Redsen	0.268±0.167	Resistant	1	0.675±0.221
F02006	0.419±0.086	Sensitive		
River John Blue	0.478±0.099	Sensitive		
08HE042-2	0.490±0.092	Sensitive		
Epicure	0.499±0.169	Sensitive		
08HE076-3	0.582±0.132	Sensitive		
British Columbia Blue	0.602±0.133	Sensitive		
08CA0623	0.626±0.165	Sensitive		
Caribe	0.668±0.257	Sensitive		
08HE077-15	0.685±0.252	Sensitive		
08CA9728-04	0.737±0.145	Sensitive		
08HE035-3	0.802±0.003	Sensitive		
08HE068-3	0.912±0.088	Sensitive		
09HE018-1	0.942±0.187	Sensitive		
Tuodu 175	0.983±0.089	Sensitive		
07HE083-15	1.105±0.108	Sensitive		
Andover	0.116±0.042	Resistant	2	0.555±0.237
08CA0687	0.132±0.004	Resistant		
F03008	0.283±0.160	Resistant		
Lenape	0.286±0.043	Resistant		
09HE028-3	0.292±0.027	Resistant		
10908-06	0.309±0.339	Sensitive		
08CA9668-24	0.385±0.101	Sensitive		
F58089	0.422±0.084	Sensitive		
Sebago	0.465±0.176	Sensitive		
F00070	0.499±0.111	Sensitive		
K113-1	0.501±0.058	Sensitive		
07HE058-5	0.507±0.157	Sensitive		
CS74109-8	0.512±0.026	Sensitive		
08HE205-1	0.525±0.013	Sensitive		
08CA0584	0.532±0.135	Sensitive		
08CA9635-19	0.577±0.164	Sensitive		
07HE032-4	0.616±0.040	Sensitive		
15113-06	0.621±0.283	Sensitive		
Zhongshu 3	0.625±0.080	Sensitive		
08HE205-3	0.654±0.112	Sensitive		

08CA0453	0.657±0.216	Sensitive		
Shepody	0.694±0.013	Sensitive		
08CA0617	0.711±0.074	Sensitive		
09HE038-5	0.748±0.171	Sensitive		
09HE031-3	0.864±0.127	Sensitive		
USDA*96-56	0.887±0.465	Sensitive		
Line 1	0.962±0.177	Sensitive		
08HE077-3	1.164±0.127	Sensitive		
AC Red Island	0.177±0.109	Resistant	3	0.565±0.270
Raritan	0.194±0.202	Resistant		
05HE5-43	0.195±0.107	Resistant		
Jizhangshu 8	0.208±0.102	Resistant		
09HE046-3	0.217±0.060	Resistant		
F01004	0.260±0.134	Resistant		
Norvally	0.277±0.078	Resistant		
Frontier Russet	0.408±0.071	Sensitive		
04HE21-11	0.413±0.243	Sensitive		
Hui-2	0.436±0.204	Sensitive		
09HE039-1	0.482±0.028	Sensitive		
N1700-6	0.504±0.101	Sensitive		
397099.4	0.578±0.169	Sensitive		
RH89-039-16	0.607±0.396	Sensitive		
09HE046-4	0.670±0.147	Sensitive		
Ranger Russet	0.68±0.166	Sensitive		
Huaen 1	0.757±0.065	Sensitive		
Pink Pearl	0.760±0.067	Sensitive		
ND6993-13	0.852±0.092	Sensitive		
AC Belmont	0.871±0.267	Sensitive		
04P48-3	0.887±0.185	Sensitive		
Purple Viking	0.897±0.223	Sensitive		
F04007	0.916±0.074	Sensitive		
F93102	0.929±0.111	Sensitive		
Alasa Frostless	0.955±0.051	Sensitive		
F66041	0.139±0.126	Resistant	4	0.529±0.255
Eshu 5	0.185±0.148	Resistant		
F58050	0.462±0.016	Sensitive		
Zhongshu 5	0.550±0.148	Sensitive		
Innovator	0.576±0.116	Sensitive		
Zhengshu 5	0.599±0.035	Sensitive		
AC Brador	0.760±0.079	Sensitive		
Favorita	0.960±0.039	Sensitive		

MPI44.1016/10	0.254±0.006	Resistant	-
07HE058-4	0.262±0.127	Resistant	-
05HE02-1	0.378±0.146	Sensitive	-
Atlantic	0.455±0.062	Sensitive	-
08CA9278-07	0.656±0.157	Sensitive	-
09HE042-2	0.671±0.217	Sensitive	-
07HE110-6	0.769±0.113	Sensitive	-
09HE038-1	0.816±0.089	Sensitive	-
ED25	0.909±0.081	Sensitive	-
Zhengshu 6	0.973±0.060	Sensitive	-
09HE011-1	1.291±0.169	Sensitive	-

[a] – means missing data as the genotype data from one marker or more of four markers were missing.

[b] The mean tuber reducing sugar content of all genotypes that have the same number of positive alleles.
