# Supplementary material for: Genetic Loci Conferring Reducing Sugar Accumulation and Conversion of Cold-Stored Potato Tubers Revealed by QTL Analysis in a Diploid Population
Source: Front Plant Sci. 2018 Mar 9;9:315. doi: 10.3389/fpls.2018.00315 (PMC5854652; doi:10.3389/fpls.2018.00315)
Supplement: Supplementary file 2 [file Table_2.PDF]

**Supplementary Table S2 Location of field trials conducted for EB population**

| Environment code | Location | Description of the location | Year |
|---|---|---|---|
| 1 | Chang Linggang | Hubei, 1700 m asl, southwest potato region | 2008 |
| 2 | Tian Chishan | Hubei, 1100 m asl, southwest potato region | 2008 |
| 3 | Tian Shui | Gansu, 1700 m asl, northwest potato region, arid | 2012 |
| 4 | Wuhan | Hubei, central potato region | 2008 |
| 5 | Wuhan | Hubei, central potato region | 2010 |
