# Supplementary material for: Genetic Loci Conferring Reducing Sugar Accumulation and Conversion of Cold-Stored Potato Tubers Revealed by QTL Analysis in a Diploid Population
Source: Front Plant Sci. 2018 Mar 9;9:315. doi: 10.3389/fpls.2018.00315 (PMC5854652; doi:10.3389/fpls.2018.00315)
Supplement: Supplementary file 3 [file Table_3.PDF]

Supplementary Table S3 QTL of CIS and REC on two parental linkage maps

Linkage map	Trait[a]	Chromosome	QTL[b]	Environment Code[c]	2-LOD interval	% Expl.[d]	Candidate Gene colocalized[e]
ED25	CIS	5	CIS_E_05-1	3	27.5-31.2	8.7	
			CIS_E_05-1	5	28.5-31.2	9.8	
	CIS	5	CIS_E_05-2	1	43.6-50.4	10.6	
	CIS	5	CIS_E_05-3	1	57.0-60.9	7.3	
			CIS_E_05-3	4	59.4-63.4	4.0	
	CIS	6	CIS_E_06-1	3	35.1-39.4	10.1	GP79_1500
			CIS_E_06-1	2	35.1-41.4	12.4	
	CIS	6	CIS_E_06-2	1	54.7-58.8	11.9	
	CIS	6	CIS_E_06-3	4	96.3-107.8	10.0	Fk_650
	CIS	7	CIS_E_07-1	1	53.7-63.8	10.6	AGPS2_250
			CIS_E_07-1	4	59.2-63.8	4.0	AGPS2_250
	CIS	9	CIS_E_09-1	3	0.0-9.2	10.1	InvGF_800
	CIS	11	CIS_E_11-1	5	70.3-77.3	9.1	
	REC	1	REC_E_01-1	2	3.0-17.9	10.8	AGPaSeS_370
	REC	2	REC_E_02-1	3	32.0-41.9	12.0	
	REC	3	REC_E_03-1	1	41.6-53.8	7.2	SSSI-11_620
	REC	5	REC_E_05-1	5	18.2-25.8	8.4	
	REC	5	REC_E_05-2	1	34.7-41.0	6.8	
	REC	6	REC_E_06-1	1	35.1-41.4	8.5	
	REC	6	REC_E_06-2	2	90.9-109.8	10.8	Fk_650
	REC	7	REC_E_07-1	3	84.1-98.8	7.8	
	REC	8	REC_E_08-1	5	50.8-53.9	7.2	

	REC	11	REC_E_11-1	4	73.3-80.6	5.2	
S. berthaultii	CIS	3	CIS_B_03-1	2	76.5-83.8	9.0	Ppe_250
	CIS	3	CIS_B_03-2	1	88.4-96.4	6.0	SbRFP1_600
	CIS	4	CIS_B_04-1	4	5.0-18.5	7.3	
	CIS	5	CIS_B_05-1	3	86.1-90.2	7.8	
	CIS	7	CIS_B_07-1	5	77.9-100	7.9	
	CIS	10	CIS_B_10-1	1	56.4-73.8	4.8	
	CIS	11	CIS_B_11-1	4	9.3-14.4	9.6	Sut1-7_1440
	CIS	11	CIS_B_11-2	1	24.3-30.5	8.6	Sut1_650
	CIS	11	CIS_B_11-3	1	64.1-85.0	6.3	
	REC	3	REC_B_03-1	2	76.5-82.8	7.0	Ppe_250
	REC	3	REC_B_03-2	1	90.5-96.4	7.3	SbRFP1_600
	REC	4	REC_B_04-1	5	57.4-59.7	4.9	
	REC	5	REC_B_05-1	4	15.5-64.4	6.6	GWD
			REC_B_05-1	5	15.5-35.8	7.5	
			REC_B_05-1	2	42.1-64.4	4.9	GWD
	REC	8	REC_B_08-1	5	74.4-79.5	6.6	
	REC	11	REC_B_11-1	1	5.3-15.4	6.8	Sut1-7_1440
	REC	11	REC_B_11-2	2	58.0-85.0	7.1	
			REC_B_11-2	1	65.1-85.0	8.9	

[a] CIS: reducing sugar content tested after storage at 4 °C for 30 d; REC: reducing sugar content tested after storage at 4 °C for 30 d and then reconditioned at 20 °C for 20 d

[b] The name of QTL includes trait name CIS or REC and the chromosome location.. E and B denote female (ED25) and male (*Solanum berthaultii* acc.) parent, respectively.

[c] Refer to Supplementary Table S2 for detail.

[d] The phenotypic variation explained by QTL.

[e] AGPaSeS and AGPS2, ADP-glucose pyrophosphorylase S, Starch synthesis; SSSI, Soluble starch synthase I, Starch synthesis; GP79, Genomic DNA fragment; Unknown; Fk, Fructokinase, Glycolysis; InvGF, Apoplastic invertase, Sucrose metabolism; Ppe, Pentose-5-phosphate 3-epimerase, Calvin cycle; SbRFP1 RING finger protein, Starch degradation; GWD, α -glucan, water dikinase, Starch degradation; Sut1 and Sut1-7, Sucrose transporter 1e, Transport (membranes).
