# Supplementary material for: Genetic Loci Conferring Reducing Sugar Accumulation and Conversion of Cold-Stored Potato Tubers Revealed by QTL Analysis in a Diploid Population
Source: Front Plant Sci. 2018 Mar 9;9:315. doi: 10.3389/fpls.2018.00315 (PMC5854652; doi:10.3389/fpls.2018.00315)
Supplement: Supplementary file 4 [file Table_4.PDF]

Supplementary Table S4 Cumulative effects of QTL in EB population

Trait[a]	No. of positive alleles	RS content (Means±SD)					No. of genotypes
		1[b]	2[b]	3[b]	4[b]	5[b]	
CIS	0	0.947±0.18	0.986±0.201	0.512±0.169	0.823±0.202	1.163±0.385	31
	1	0.842±0.264	0.932±0.214	0.492±0.223	0.806±0.205	1.219±0.331	63
	2	0.827±0.219	0.927±0.214	0.358±0.153	0.798±0.232	1.058±0.384	44
	3	0.714±0.196	0.838±0.218	0.329±0.177	0.717±0.177	1.029±0.331	26
	4	0.600±0.162	0.743±0.187	0.400±0.159	0.608±0.165	0.876±0.332	8
REC	0	0.545±0.201	0.578±0.172	0.289±0.168	0.533±0.168	0.754±0.290	51
	1	0.469±0.202	0.514±0.166	0.253±0.153	0.436±0.161	0.666±0.263	89
	2	0.424±0.164	0.443±0.139	0.250±0.238	0.385±0.147	0.682±0.278	36

[a] CIS: reducing sugar content tested after storage at 4 °C for 30 d; REC: reducing sugar content tested after storage at 4 °C for 30 d and then reconditioned at 20 °C for 20 d.

[b] Environment code, refer to Supplementary Table S2 for detail.
