# Supplementary figures and images for: Genetic Loci Conferring Reducing Sugar Accumulation and Conversion of Cold-Stored Potato Tubers Revealed by QTL Analysis in a Diploid Population
Source: Front Plant Sci. 2018 Mar 9;9:315. doi: 10.3389/fpls.2018.00315 (PMC5854652; doi:10.3389/fpls.2018.00315)

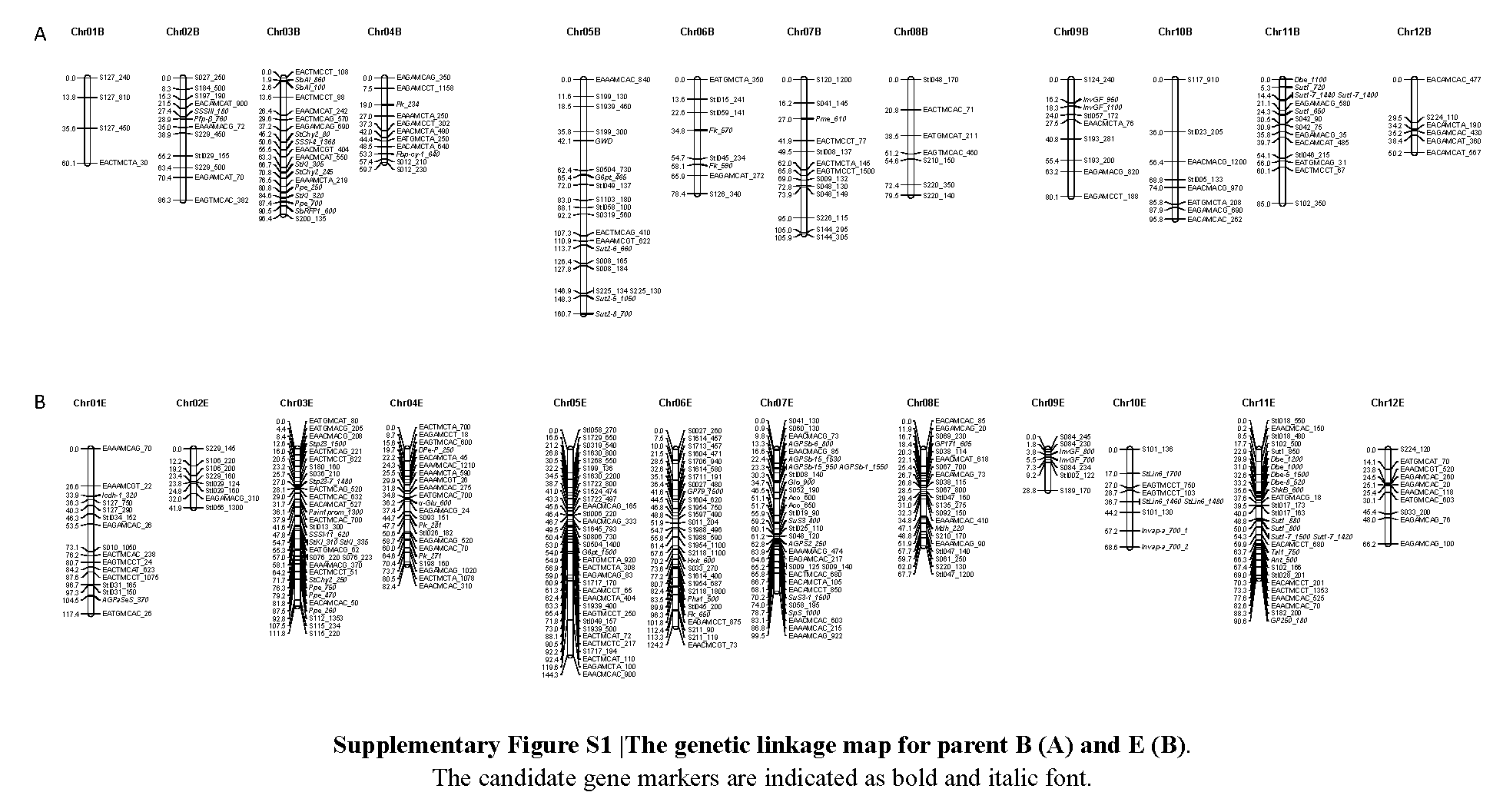

Supplement: Supplementary file 5 [file Image_1.TIF]
